# Enhancing Mental Health and Medication Adherence Among Men Who Have Sex With Men Recently Diagnosed With HIV With a Dialectical Behavior Therapy–Informed Intervention Incorporating mHealth, Online Skills Training, and Phone Coaching: Development Study Using Human-Centered Design Approach

**DOI:** 10.2196/47903

**Published:** 2023-10-13

**Authors:** Liying Wang, Weichao Yuwen, Wenzhe Hua, Lingxiao Chen, Vibh Forsythe Cox, Huang Zheng, Zhen Ning, Zhuojun Zhao, Zhaoyu Liu, Yunzhang Jiang, Xinran Li, Yawen Guo, Jane M Simoni

**Affiliations:** 1 Department of Psychology University of Washington Seattle, WA United States; 2 School of Nursing & Healthcare Leadership University of Washington, Tacoma Tacoma, WA United States; 3 School of Nursing Shanghai Jiao Tong University Shanghai China; 4 Department of Human and Organizational Development Vanderbilt University Nashville, TN United States; 5 Shanghai China Sex Worker & Men who have Sex with Men Center Shanghai China; 6 Department of HIV/STD Control and Prevention Shanghai Municipal Centers for Disease Control and Prevention Shanghai China; 7 Department of Human-Centered Design and Engineering University of Washington Seattle, WA United States; 8 Courant Institute of Mathematical Sciences New York University New York, NY United States; 9 Information School University of Washington Seattle, WA United States

**Keywords:** intervention mapping, participatory approach, cultural adaptation, dialectical behavior therapy, DBT, men who have sex with men, MSM, coping skill training

## Abstract

**Background:**

Mental health problems are common among men who have sex with men (MSM) living with HIV and may negatively affect medication adherence. Psychosocial interventions designed to address these urgent needs are scarce in China. Incorporating behavioral health theories into intervention development strengthens the effectiveness of these interventions. The absence of a robust theoretical basis for interventions may also present challenges to identify active intervention ingredients.

**Objective:**

This study aims to systematically describe the development of a mobile health–based intervention for MSM recently diagnosed with HIV in China, including the theoretical basis for the content and the considerations for its technological delivery.

**Methods:**

We used intervention mapping (IM) to guide overall intervention development, a behavioral intervention technology model for technological delivery design, and a human-centered design and cultural adaptation model for intervention tailoring throughout all steps of IM.

**Results:**

The dialectical behavior therapy (DBT)–informed intervention, *Turning to Sunshine*, comprised 3 components: app-based individual skills learning, group-based skills training, and on-demand phone coaching. The theoretical basis for the intervention content is based on the DBT model of emotions, which fits our conceptualization of the intervention user’s mental health needs. The intervention aims to help MSM recently diagnosed with HIV (1) survive moments of high emotional intensity and strong action urges, (2) change emotional expression to regulate emotions, and (3) reduce emotional vulnerability, as well as (4) augment community resources for mental health services. Technological delivery considerations included rationale of the medium, complexity, and esthetics of information delivery; data logs; data visualization; notifications; and passive data collection.

**Conclusions:**

This study laid out the steps for the development of a DBT-informed mobile health intervention that integrated app-based individual learning, group-based skills training, and phone coaching. This intervention, *Turning to Sunshine*, aims to improve mental health outcomes for MSM newly diagnosed with HIV in China. The IM framework informed by human-centered design principles and cultural adaptation considerations offered a systematic approach to develop the current intervention and tailor it to the target intervention users. The behavioral intervention technology model facilitated the translation of behavioral intervention strategies into technological delivery components. The systematic development and reporting of the current intervention can serve as a guide for similar intervention studies. The content of the current intervention could be adapted for a broader population with similar emotional struggles to improve their mental health outcomes.

## Introduction

### Background

Approximately 38.4 million people worldwide live with HIV [1]. Men who have sex with men (MSM) in China shoulder a substantial burden of HIV and experience a higher prevalence of depression, anxiety, and suicidal ideation compared with HIV-negative MSM [2,3]. Emotional distress is especially prominent among those who have been recently diagnosed (within the previous year) [4]. However, China is not equipped to address this tremendous demand—for 100,000 people, there were only 2.20 psychiatrists and 5.42 mental health nurses according to a report by the World Health Organization in 2019 [5]. Digital health technologies (mobile health [mHealth]) can potentially increase the accessibility of mental health interventions [6]. mHealth psychosocial interventions hold promise for improving mental health among the general population [7], people living with HIV [8], and MSM living with HIV [9]. mHealth interventions are especially suitable in a setting with limited mental health resources such as China, especially given the high digital literacy among the Chinese population [5]. However, few mHealth intervention studies have been conducted among people living with HIV in China to improve their mental health outcomes and medication adherence. Most existing mHealth interventions for people living with HIV in China target HIV self-testing [10] and HIV transmission risk [11]. The primary goal of this study was to describe the development of a novel mHealth intervention for MSM recently diagnosed with HIV in China to improve their mental health and medication adherence, with an emphasis on theory-based development.

Health behavior theories and models play an important role in guiding the development of behavioral health interventions [12]. Theory-based behavioral interventions overall are more effective in leading to behavior change compared with those without a theoretical basis [13]. In addition, the effectiveness of theory-based interventions can be further enhanced by using evidence-based behavior change techniques that are supported by the literature [14]. However, the links between theory, behavior change techniques, and intervention design are often not reported in detail [15]. The lack of a detailed rationale for intervention components limits our ability to analyze the active ingredients of interventions, improve future intervention design, and adapt intervention programs for a different group of users or cultural context. Intervention mapping (IM) offers an overarching framework for the systematic development and reporting of behavioral health interventions. The IM framework outlines 6 steps for developing an intervention, from the logic model of the problem to an evaluation plan for the intervention. It starts with a needs assessment step to understand the target intervention users while considering behavior change theories to guide behavior change methods and practical strategies [16]. The framework has an innate flexibility to incorporate theories, approaches, and empirical evidence that contribute to the overall intervention goals.

Behavioral health interventions delivered on mobile platforms (mHealth) such as mobile apps have the advantage of higher accessibility and potential for scalability, with the flexibility to be used independently or adjunct to other health services [17]. In addition to the theoretical basis and evidence-supported behavior change techniques of the intervention content, the effectiveness of mHealth interventions also hinges on careful consideration of the mode of delivery [18]. The technological delivery of behavioral health interventions goes beyond digitalizing the content of a psychosocial intervention. It requires careful consideration of the technical implementation of design components for software functionality to help users reach their behavior change goals [18]. The behavioral intervention technology (BIT) model proposed by Mohr et al [19] filled this critical gap in mHealth research by outlining the technological considerations (eg, elements, characteristics, and workflow) to guide the development of an architecture that fits the users’ needs, capabilities, and preferences. For example, the medium of information delivery should be carefully chosen to maximize the intervention effect considering the fit between the medium type and the users. The text format allows the user to control their speed of processing the information, whereas the complexity limits it to more educated users [20]. Notification messages can serve as reminders for users to engage in certain activities (eg, exercise). The visualization of mood in a line chart may increase users’ awareness of their emotions, which is the first step toward using emotion regulation skills early and effectively. A persuasive system design uses primary task support (eg, simplifying complex behaviors and self-monitoring), dialogue support (eg, positive reinforcement), credibility, and social networking components to assist behavior change, maintenance, and adherence to the mHealth intervention [21]. However, the literature on mHealth behavioral interventions mostly focuses on the content of the behavior change intervention without a description of the rationale behind the technical delivery of the content [12,22].

The tailoring and adaptation of behavior change techniques and delivery to the target population is a critical step in the development of mHealth interventions. A community-engaged research approach harnesses the expertise of community stakeholders and emphasizes mutual learning and shared decision-making between the community and academic researchers. In health disparities research, this approach enables researchers to gain a deeper understanding of the determinants of health at individual and contextual levels [23]. In digital health research, human-centered design (HCD) principles prioritize the user experience when interacting with a digital system with the aim of creating effective, efficient, and engaging products that are easy to learn. The creation of such products is grounded in an in-depth understanding of the user and the context using methods such as co-design sessions, usability tests, and rapid prototyping and iterative development [24]. With their unique focus on user experience, HCD principles and methods are increasingly being applied to tailor psychosocial interventions to the users to create useful, usable, valuable, desirable, credible, and accessible intervention programs.

Contextual factors need to be considered early in the formative stage of intervention development to inform adaptation and tailoring to the target population [25]. The field of mental health interventions increasingly realizes the importance of culturally responsive interventions [26]. Culturally adapted interventions were more effective in improving mental health outcomes among Black and ethnically diverse individuals compared with unadapted active interventions [27]. According to the ecological validity framework (EVF), cultural adaptations of mental health interventions include the following dimensions: language, persons assisting with intervention delivery, metaphors, content, concepts, client goals, and behavior change methods.

### Objectives

In this paper, we illustrate (1) the steps of IM that guided the development of the current intervention; (2) the design of technological features for delivery components using the BIT model; and (3) how content and delivery were tailored using HCD principles, cultural adaptation of evidence-based psychotherapy, and a community-based participatory approach.

## Methods

### Overview

This section focuses on reporting the theory-based rationale underlying the intervention development process. The details of co-design and usability testing are mentioned when relevant to the intervention rationale. A full report of the procedure and results of the co-design and usability testing sessions is underway and will be published elsewhere.

### Intervention Development Using IM

The intervention development followed the steps outlined in the IM protocol, including step 1—conducting a needs assessment, step 2—determining the intervention goals and objectives, step 3—selecting intervention and behavior change methods, and step 4—designing and developing program materials [16].

Needs assessment was the first step of IM. We conducted one-on-one stakeholder interviews with MSM living with HIV (n=15), staff at Shanghai Municipal Center for Disease Control and Prevention (SCDC; n=4), and Shanghai China Sex Worker and MSM Center (SCMC; n=5) to identify unmet mental health needs guided by stress and coping theory as well as a socioecological framework. The inclusion criteria for MSM living with HIV were as follows: (1) being aged ≥18 years, (2) being HIV positive, (3) having engaged in sex with a man in their lifetime, (4) having already started antiretroviral therapy, and (5) having access to the internet. Moreover, participants filled out the Patient Health Questionnaire–9 and were included if their score was <15 (minimal to moderate depression). The inclusion criteria for staff members at SCDC and SCMC were as follows: (1) being aged ≥18 years and (2) working with individuals living with HIV.

The interview questions are presented in Textbox 1. More details regarding the needs assessment are reported elsewhere (L Wang, unpublished data, July 2023). Specifically, we assessed stress and coping stress following an HIV diagnosis, the individual coping process, and community resources (L Wang, unpublished data, July 2023). We used stress and coping theory and a socioecological framework to guide the interviews and qualitative data analysis.

**Interview questions for needs assessment.**

- Tell me about the process of getting the diagnosis. How did you feel the moment you received the positive diagnosis? How did you contract HIV? Who informed you? How did they inform you? How did you react? How did that make you feel?
- How has HIV impacted your life, especially in the first few months of receiving the diagnosis? How was your mood and emotional status overall? What did you do to feel better? How did it work out (helpful or not helpful)?
- Describe problems or difficult situations related to HIV. Tell me how you approached it, what happened next, and what was the outcome? How did you feel about the outcome?
- What support have you received from Shanghai China Sex Worker and Men Who Have Sex With Men Center? What support did you not get but would like to have?
- Looking back, is there anything that you wish you had done differently to deal with the situation better?

An intervention matrix was developed as a result of step 2 to represent the overall program goals (ie, the results the intervention intends to achieve), performance objectives (ie, the actual behaviors each intervention user needs to perform to achieve the results), and change objectives (ie, the behavioral determinants that need to change for the intervention user to perform the desired behaviors). In the intervention matrix, the performance objectives in each row intersected with the behavioral determinants in each column to generate change objectives in each cell. The performance objectives were selected based on needs assessment results and relevant theories (ie, the dialectical behavior therapy [DBT] model of emotion). The selection of behavioral determinants was guided by the model of behavior (Capability, Opportunity, and Motivation–Behavior [COM-B]) and behavior change models (Behavior Change Wheel [BCW]), as well as the relevance of the constructs to this study [28]. The intervention matrix outlined the logic model of change and represented the active ingredients of the intervention program.

### Technological Delivery Using BIT

The BIT model aims to facilitate the design of technological features that maximize the impact of the behavioral intervention. This framework integrates the theoretical why (ie, aims and goals of the intervention) and how (ie, conceptual behavior change strategies) with the technological what (ie, technological elements), how (ie, medium and esthetics), and when (ie, workflow). This study used the BIT framework to promote the synergistic relationship between intervention content, delivery methods, and technological design. The integration of IM and BIT in this study aimed to translate the intervention objectives and strategies into app features by guiding design considerations of the digital delivery platform.

### Intervention Tailoring

#### HCD Principles and Strategies

HCD places users’ needs and preferences at the center of attention when designing a service or product, drawing from human-computer interaction, service design, and cognitive psychology. Other important HCD principles include (1) involving users in the design process and critical design decision-making, (2) aligning the product with users’ needs, and (3) incorporating user feedback into the design-prototyping iterative process to improve user experience gradually [29]. The general goal of applying HCD principles is to generate products that fit individual needs and preferences and contextual constraints to optimize effectiveness and efficiency in helping the user accomplish a particular goal in a satisfactory manner [30]. HCD principles have been applied to improving the user experience with software and physical products and optimize health care service delivery [31,32]. Recognizing its potential to improve the development and implementation of psychosocial interventions, researchers have identified HCD strategies to use when translating behavioral health research and evidence-based practices (eg, evidence-based psychotherapy) to health services across different settings [29,33].

For the purpose of this project, we used the following HCD strategies to tailor our intervention strategies to users. Table 1 presents the strategies and applications used in this study. Specifically, we used co-design sessions (n=10) and usability testing (n=15) to tailor the intervention and iteratively redesign it. Web-based co-design sessions were conducted, where we used Mural (LUMA Institute, LLC) and BoardMix (Boyun Technology Limited) to facilitate live collaboration. Each co-design session included 2 research team members and 2 study participants. One researcher led the co-design session, whereas the other asked follow-up questions and took notes when needed. Co-design sessions were designed based on the results from the needs assessments and included two sections: (1) content design to further prompt potential users for their experience of emotional struggles and commonly used coping strategies after HIV diagnosis and (2) delivery design to generate ideas for a potential intervention or service that could facilitate coping. Usability testing sessions involved a think-aloud method to examine the overall ease of use of the main app features (eg, medication and mood tracking) and esthetics (eg, color palette).

**Table 1:** Human-centered design (HCD) strategy definitions and application in this study.

HCD strategies			Definition		Application in this study
**Formative evaluation**					
	Interviews	Use individual interviews with potential users to collect information regarding user preferences, experiences, and priorities.		Needs assessment interviews with MSM^a^ living with HIV, SCMC^b^ staff, and SCDC^c^ staff to identify mental health needs and service gaps.	
	Define target users and their needs	Identify and develop a list of target problems based on the input from those who are affected by the problem.		On the basis of the needs assessment results, define psychological difficulties after receiving HIV diagnosis, their coping process, and the unmet mental health needs.	
**Design-focused techniques**					
	Co-design sessions	Designers, potential users, and stakeholders collaboratively design an intervention prototype.		MSM living with HIV, therapists, and SCMC and SCDC staff meet to design and tailor the content and delivery of the coping skills intervention.	
	Passive storyboards	End users are first presented with scenarios and prompted regarding how they would navigate the scenario.		During the co-design sessions, MSM living with HIV were presented with likely scenarios after receiving the diagnosis and asked about the strategies they used to cope with the emotions.	
	Use associative object-based techniques	Ask the users to sort, rank, or organize solutions to a problem or other constructs relevant to the intervention.		During the co-design sessions, MSM living with HIV were asked to conceptually group coping strategies, match them with intensity of emotions, and rank their importance and feasibility.	
	Parallel prototyping	Multiple design concepts were implemented and tested out to select the best solution.		Multiple designers created a podcast page independently following a set of agreed-upon principles to diversify design solutions.	
	Engage in cycles of rapid prototyping and iterative development	Start with a simple prototype (wireframe) and use it to obtain feedback from users and improve the design and development.		During usability testing, we used a wireframe app prototype to obtain a few rounds of feedback from stakeholders and iteratively improved and built a high-fidelity design.	
**Summative evaluation**					
	Think-aloud exercises	The end users speak aloud when completing a task using the app.		In usability testing sessions, we used this technique to understand user interaction with the app.	
	Heuristic evaluation	Researchers and designers evaluate the prototypes guided by heuristics.		We consulted domain experts to examine the usability of the intervention materials and protocols.	
	Interpretation sessions and presenting user reports to stakeholders	Summarize the results from the interview and co-design sessions and present them to stakeholders.		The results from the needs assessment were presented to SCDC and SCMC staff and participants in the co-design sessions.	

^a^MSM: men who have sex with men.

^b^SCMC: Shanghai China Sex Worker and MSM Center.

^c^SCDC: Shanghai Municipal Center for Disease Control and Prevention.

#### Cultural Adaptation

Cultural adaptation of evidence-based treatment or intervention refers to modifications of the protocol to improve the compatibility of the treatment or intervention with individuals’ cultural background, which includes but is not limited to cultural values, practices, beliefs, and preferences [34]. The EVF offers practical guidance by outlining the 8 dimensions to consider in the cultural adaptation of evidence-based treatment or interventions, including language, persons, metaphors, content, concepts, goals, methods, and context [35].

The adaptation of evidence-based psychotherapy techniques considers therapist characteristics (eg, ethnicity and language), therapeutic elements (eg, culturally sensitive materials), and delivery setting (location and method of access) [27]. Most psychosocial interventions and evidence-based psychotherapies were developed and tested in a context characterized by the following features: Western, educated, industrialized, rich, and democratic [36]. Thus, the content and approaches might not be culturally responsive for individuals with diverse racial and ethnic backgrounds. Although psychotherapy is idiographic to the individual in nature, therapeutic elements such as culturally relevant metaphors could either greatly facilitate or impede the therapeutic process [34].

#### Community-Based Participatory Approach

This work was guided by community stakeholders and expert consultation. The first author, a predoctoral trainee in clinical psychology, led the intervention development with extensive engagement with community partners and consultation with content experts. We established partnerships with SCDC and SCMC to gain a deeper understanding of the community’s needs and priorities. SCDC and SCMC collaboratively provide services such as postdiagnosis consultation, in-person gatherings, and phone call follow-ups to assist individuals in transitioning to living with HIV. The researchers drew upon input from community partners in project planning, conceptualization, data collection, and manuscript preparation. One SCDC and 1 SCMC staff member were consulted through biweekly meetings throughout the study period to seek feedback and make timely adjustments to the study protocol. The intervention content outline was first presented to our SCDC and SCMC partners, after which SCMC staff (n=6) were gathered for a feedback session after the intervention content was created. The details are reported elsewhere.

### Ethical Considerations

All study participants in the needs assessment, co-design sessions, and usability testing provided informed consent before enrollment in the study. Data were deidentified to protect participants’ privacy. All participants received compensation (20 USD) for their time and contribution to the study. This study was reviewed and approved by the institutional review board of the University of Washington (STUDY00011948).

## Results

### Overview

We describe the development of the current mHealth intervention following the four steps outlined in the IM framework: (1) conducting a needs assessment, (2) determining intervention goals and objectives, (3) selecting intervention and behavior change methods, and (4) designing and developing program materials. The results of each IM step also incorporated insights obtained from HCD principles and cultural adaptation dimensions to demonstrate the rationale behind intervention tailoring. In IM step 4, the BIT model was presented to describe the technological delivery of the intervention.

### Step 1: Needs Assessment

Overall, we found that stress from multiple sociological levels interacted with each other and produced compounded effects on the psychological struggles among MSM living with HIV. MSM living with HIV reported being overwhelmed by emotional turbulence upon receiving a positive diagnosis. They reported prolonged distressing HIV-related thoughts and emotions as they adjusted to living with HIV. However, these mental health needs were largely unmet and sometimes led to destructive and maladaptive behaviors (eg, suicidal ideation and substance use) and prolonged distress (eg, depression and anxiety). Individuals lacked the skills to regulate distressing emotions and thoughts and relied heavily on close relationships for support. Those who did not have support from friends or family mainly used self-help books and mental health apps (eg, Headspace) to cope with their emotional pain. Individuals reported a lack of mental health support from the community organization. Services at SCMC and SCDC focused on linkage and engagement with HIV-related care. The staff provided occasional check-ins and organized peer support groups but felt ill-equipped to provide appropriate mental health services. Both MSM living with HIV and staff at SCDC and SCMC recognized the mental health needs and the service gap.

On the basis of the needs assessment results, implications for multiple-level intervention development were reported by L Wang (unpublished data, July 2023), including (1) individual coping skill training on emotional difficulties (initial high-intensity emotions and chronic emotional distress) to improve mental health outcomes, (2) peer or lay provider training to increase community mental health resources and support, (3) leveraging role models to reduce internalized HIV-related stigma, and (4) disclosure interventions to increase friend and family support.

### Step 2: Intervention Goals and Objectives

The intervention goals were determined based on the results of the needs assessment. The intervention objectives were then specified using relevant theories and results from the co-design sessions.

#### Overall Program Goals

The program goals are listed in the first column of Table 2. Informed by the needs assessment results, the first overall goal of the intervention program was to target MSM recently diagnosed with HIV to enhance overall mental health. Another goal of this program was to promote community and structural mental health support. MSM newly diagnosed with HIV face double stigma, which causes them to isolate themselves and not seek help. SCMC staff are usually the first point of contact for MSM newly diagnosed with HIV, which places them at a unique position to provide first aid emotional support. SCMC staff could also provide long-term emotional support by incorporating mental health check-ins into their current follow-up calls that are focused on medication adherence.

**Table 2:** The matrix of change objectives.

Program objectives	Performance objectives	Intervention functions and theoretical behavior change methods		
		Education	Skills training (DBT^a^ skills)	Enablement and modeling
Mental health (objective 1)	Survive moments of high emotional intensity and strong action urges	Emotional intensity and impulsive behavior	How to get through a crisis and overwhelming emotions without making things worse	SMS text message or phone coaching from SCMC^b^ staff
Mental health (objective 2)	Change the emotional expression to regulate emotions	What are emotion and the functions of emotion	How to regulate emotions that are not effective given a certain goal	Role model; peer support group; and goal setting, monitoring, and feedback
Mental health (objective 3)	Reduce emotional vulnerability	What is emotional vulnerability and why it matters	How to build emotional resilience and reduce vulnerability to the emotional mind	Role model; peer support group; and goal setting, monitoring, and feedback
Community capacity building	Promoting community and structural mental health support	DBT model of emotion and DBT skills	Basic behavioral therapy techniques to reinforce skills learning; phone coaching skills	Group supervision

^a^DBT: dialectical behavior therapy.

^b^SCMC: Shanghai China Sex Worker and Men Who Have Sex With Men Center.

#### Performance Objectives

Performance objective refers to the behaviors that each intervention user needs to perform to reach the overall program goal. We modified this definition to incorporate the community organization given the existing service structure and the importance of community support to MSM recently diagnosed with HIV. The results from the needs assessment, the DBT model of emotions, and co-design sessions informed the identification of performance objectives (Textbox 1).

**Figure 1:**
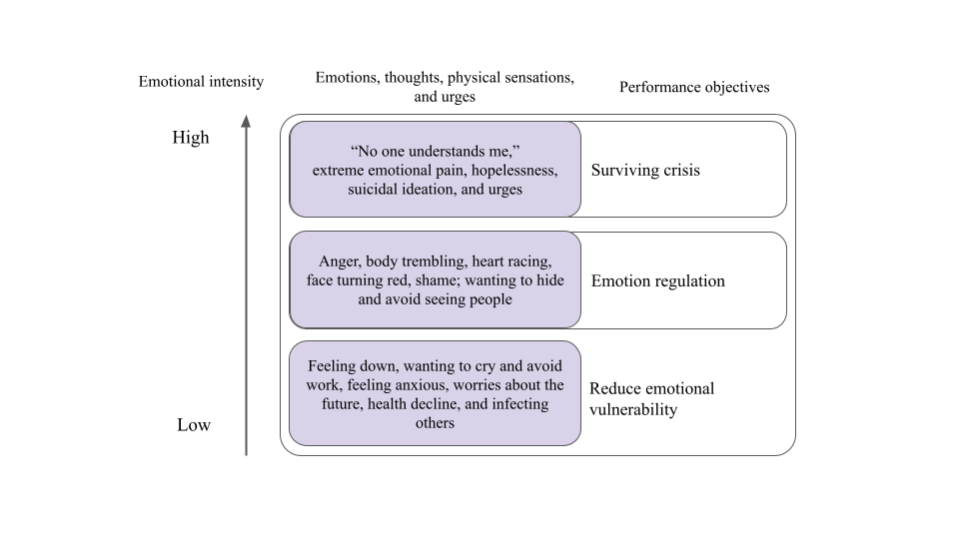
Emotional intensity and performance objectives.

The needs assessment revealed the emotional struggles among MSM living with HIV, with intense emotions immediately after diagnosis and chronic emotional distress. Figure 1 shows the emotional difficulties reported by MSM living with HIV. The initial unbearable emotional suffering led to extreme thoughts and urges such as suicidal ideation and attempts among several participants. The chronic emotional difficulties impaired social functioning and led to avoidance of daily activities such as work and socializing. Emotion dysregulation is a risk factor for developing mental health problems through interaction with impulse control and interpersonal difficulties [37].

Recognizing the difficulties regarding coping with emotions and their implications for long-term mental health, we decided to use the DBT model of emotions to guide our understanding of emotions and identify behaviors that individuals can perform to address emotion regulation difficulties (ie, performance objectives). The DBT model of emotions offers a framework to understand emotional responses and outlines concrete skills to regulate emotions to reduce the risk of developing mental health problems, decrease their negative consequences, and improve global functioning [37,38].

According to the DBT model of emotions, emotional responses are systemic responses that include the following subsystems: (1) emotional vulnerability, (2) prompting events (ie, internal or external events as emotional cues), (3) interpretation of the prompting events, (4) biological changes and experience of emotions (eg, action urges), (5) verbal and nonverbal expression of emotions, and (6) aftereffects of emotional response (eg, secondary emotions). The subsystems are interactive so that the overall emotional response can be modified by changing 1 or more of the components of the system.

The co-design sessions further revealed that the number of coping strategies for high–emotional intensity situations (n=19) was smaller compared with that of strategies for lower–emotional intensity situations (n=26). In addition, substance use, such as smoking and drinking alcohol, emerged as a coping strategy, especially for situations with high emotional intensity. For example, in one of the co-design sessions, both participants mentioned that they drank when they felt overwhelmed by high-intensity emotions. Regardless of the emotional intensity levels, the most commonly used strategies by participants were distraction (eg, keeping busy with work or other activities), talking with trusted friends or family, problem-solving, and exercise. Spending time alone was the most frequently mentioned coping strategy when they felt overwhelmed by emotions. In summary, the co-design sessions suggested limited skills regarding emotion regulation, especially skill deficits in coping with high emotional arousal.

On the basis of these results, we selected three components from the DBT model of emotion to derive performance objectives: (1) biological changes and experience of emotions (eg, action urges), (2) verbal and nonverbal expression of emotions, and (3) emotional vulnerability given its broad implications for other subsystems. Figure 1 presents the performance objectives mapped onto the difficult emotions uncovered from the needs assessment sessions. The performance objectives are outlined in Textbox 2.

**The performance objectives corresponding to the 3 selected components from the dialectical behavior therapy model of emotion.**

**Survive moments of high emotional intensity and strong action urges**

- Emotions are accompanied by biological changes and action urges. For example, fear is often associated with the activation of the sympathetic nervous system, which increases our heart rate, leads to sweating, enlarges the size of our pupils, and leads to the active urge to fight or flight. In a crisis situation, such as the moment of receiving the positive diagnosis, the emotional intensity is high, and the emotional pain can be overwhelming. The related performance objective is to survive the crisis and tolerate the pain and distress without acting on destructive urges (eg, suicide).

**Change emotional expression to regulate emotions**

- Emotional expression includes facial expressions, body language, and behaviors. For example, sadness is often expressed through eyes dropping, not smiling, sobbing, and a quiet and monotonous voice.
- Changing emotional expression can lead to changes in emotions as well. When the emotional intensity is not as extreme, such as when taking medication every day reminds individuals of their diagnosis, the effective use of emotion regulation skills can help reduce emotional pain. The related performance objective is to change emotional expression to regulate emotions [37].

**Reduce emotional vulnerability**

- Vulnerability factors increase individuals’ sensitivity to a prompting event, may lead to biased interpretations of the event, and may increase the intensity of biological reaction and expression. Vulnerability factors may include lack of sleep, hunger, influence of substances (eg, alcohol and mood-altering drugs), or a series of negative life events. When these vulnerability factors are modified, the emotional response or the intensity of that response can change. Thus, the related performance objective is to reduce overall emotional vulnerability.

Regarding the program goal of promoting community and structural mental health support, the performance objective was to create a structured environment to support individuals’ learning. Learning behavioral skills is especially challenging when the person does not receive reinforcement from a supportive environment for such learning [37]. In mHealth interventions, periodic human support in general improves adherence to mHealth tools and is linked to better outcomes. Supportive accountability is a mechanism that contributes to the coaching effect by providing assistance in clarifying goals, monitoring, and reviewing progress [39]. Therefore, the performance objective at the structural level is to complement individual skills learning with community services (eg, skills training group) informed by the guidelines and principles of the DBT Skills Training Manual [37].

In summary, to reach the overall program goal, the individual- and community-level performance objectives need to be attained, as listed in the second column of Table 1.

### Step 3: Intervention and Behavior Change Methods

We determined the change objectives of the intervention program based on the aforementioned performance objectives and the behavioral determinants delineated by the COM-B model of behavior, including capability, motivation, and opportunity [28]. Table 3 shows the components of the COM-B model, definitions, and intervention functions applied in this study.

**Table 3:** Determining change objectives using links between components of the Capability, Opportunity, and Motivation–Behavior (COM-B) model and intervention functions relevant to this study.

Behavior determinants (COM-B)	Definition and example application in this study	Intervention functions and theoretical behavior change methods			
		Education	Training	Enablement	Modeling
Capability	Individual’s psychological and physical capacity to perform a certain behavior (eg, learning the necessary emotion regulation skills)	✓	✓	✓	✓
Opportunity	Nonindividual factors that prompt or facilitate the desired behaviors, including physical and social opportunity		✓	✓	✓
Motivation	Intentional processes that direct and induce behavior, including both conscious decision-making (ie, reflective motivation) and habitual process and emotion-driven behaviors (ie, automatic motivation), for example, gaining motivation through observing successful application of coping skills by peers	✓	✓		✓

The COM-B model connects the model of behavior directly with intervention functions that can target one or more behavioral determinants through the BCW [28]. Thus, the COM-B model combined with the BCW offers an efficient method to determine intervention functions based on behavioral targets. In addition, contextual factors are inherent components of this framework, reflected in physical and social opportunity. Given the dependence of MSM recently diagnosed with HIV on community resources, contextual intervention is especially appropriate to induce and maintain behavior change to reach the program’s performance objectives. The automatic processing element within the motivation component of the COM-B system aligns well with the findings from the needs assessment, which indicate the benefits of peer role models in coping with internalized stigma and generating hope (L Wang, unpublished data, July 2023).

Selecting appropriate intervention functions to fit the characteristics of the target population and contextual features could optimize the effectiveness of the intervention [40,41]. Thus, we selected the four most relevant and feasible intervention functions that target all 3 components in the COM-B model of behavior (Table 2):

1. Education: knowledge and understanding of emotion can increase psychological capability, and the rationale behind and benefits of emotion regulation can enhance the reflective motivation for the participants to learn skills.
2. Training: learning and practicing cognitive and behavioral skills both individually and in a group setting can help participants gain psychological capability.
3. Enablement: a skills group and SCMC phone coaching can guide the participants to select and practice appropriate skills, especially in a situation with high emotional arousal; in addition, staff and app-assisted goal setting, monitoring, and timely feedback are reinforcers of behavior change.
4. Modeling: the skills group training environment provides ample opportunities for imitative learning and role modeling.

In summary, the intervention will use education, skills training, enablement, and modeling to target the capability, motivation, and opportunity of behaviors to reach the performance goals described in the previous step (Table 2).

### Step 4: Design and Development of Program Materials

#### Overview

The intervention program includes 3 components: individual skill learning using the mobile app, accompanied by a skills group, and on-demand phone coaching. This overall program design is informed by the results from co-design sessions and the elements of DBT treatment programs.

The rationale for each intervention component is as follows:

1. App-based individual skills learning: emotion regulation and coping skills were highlighted by the participants regarding the content of the program that they would like to receive. They preferred the content to be tailored to the needs of the HIV-positive community. They emphasized that the content needs to have credibility while using language that could be easily understood by a general public without much mental health background. Each individual skills-learning module is followed by a quiz to reinforce learning and increase engagement.
2. Group-based online skills training facilitated by SCMC staff: the skills training group was perceived by participants as efficient in providing low-intensity mental health support in which the shared experience was perceived to increase their ability to empathize with one another. SCMC staff has established trusting relationships with HIV-positive MSM and was perceived as a trusted resource for HIV-related information. Compared with in-person groups, web-based groups have the unique advantage of protecting participants’ privacy.
3. On-demand phone coaching: phone coaching was incorporated to meet higher needs for mental health support, especially in the early stages after HIV diagnosis.

The cultural adaptation of the DBT skills was based on the EVF, including the following dimensions: language, persons, metaphors, content, concept, goals, method, and context [34]. For example, the language translation of certain DBT terms emphasized conveying the meaning accurately rather than word-to-word translation. Radical acceptance DBT, which refers to complete acceptance of reality, was translated as “全然” (*quan ran*) accordingly rather than being translated verbatim as “全然” (*ji jin*), which means extreme. We also used culturally relevant metaphors, stories, and skill practice examples that resonated with HIV-positive individuals to design the intervention content. Examples of adaptations in other dimensions are shown in Table 4.

**Table 4:** Cultural adaptation domains and applications.

Domain	Definition	Application in this study
Language	Translate the language so that the intervention can be understood by the users	DBT^a^-based skills content was all translated into Mandarin. Translations of certain DBT terms were adjusted to convey their original meaning rather than adopting a direct verbatim translation. For example, “radical” was translated as “全然” (*quan ran*) rather than “激进” (*ji jin*) to convey the complete acceptance of reality.
Persons	Who would deliver the intervention	Staff members at SCMC^b^ received training to facilitate the skills group and provide phone coaching. SCMC staff have long-standing trusting relationships with the HIV-positive MSM^c^ community and expertise in HIV-related treatment orientation and can provide a stable point of contact for continued mental health support after HIV diagnosis.
Metaphors	Use metaphors that are close to the target culture to assist understanding	We used a cultural idiom (“情绪决堤,” qing xu jue di, meaning emotion is like water flooding over the dam) to describe how a lack of emotional resilience and coping skills is like having a lower dam so that, whenever a negative event in life causes a wave, the emotion will flood over the dam.
Content	Adjustments made to content to meet the specific cultural and intervention context	The podcast was designed so that the content would resonate with the user while not using terms such as “HIV positive.” For example, we use a terminal diagnosis of a family member as an example event to illustrate radical acceptance skills, which share some similarities with the coping process after HIV diagnosis. This is to protect users’ privacy in case others happen to hear the content of this podcast. This can also reduce the potential barrier of shame and internalized stigma regarding accessing this content.
Concept	Tailor the therapy’s relevant concepts to aid understanding in the given context	We used “举杯消愁愁更愁” (“drinking to reduce sadness only does the opposite”), a line from a well-known poem, to express the concept of ineffective coping with negative mood using substances such as alcohol.
Goals	Align the goals of the intervention or treatment with the target users	On the basis of the results of the needs assessment, we define the main goal of the intervention to be providing skills to relieve emotional pain that might get in the way of participants adhering to HIV-related treatment, staying engaged with social life, and continuing to lead a life worth living.
Method	Adapt the format of the intervention to fit the context of delivery	We decided to add the group-based online skills training to (1) provide a peer-supported learning environment to cope with difficult emotions, (2) learn from peers’ successful coping stories and journeys to reduce the internalized stigma of HIV, and (3) protect individuals’ privacy using a web-based format.
Context	Consider the broader contextual constraints and factors that might hinder or facilitate intervention delivery	We used the term “skills training class” and “skills training app” rather than terms related to mental health considering the stigma related to mental health conditions. Similarly, we did not use any terms that explicitly link the app content to HIV-positive individuals considering the social impact that accidental disclosure may have on individuals, such as rejection by family and loss of employment.

^a^DBT: dialectical behavior therapy.

^b^SCMC: Shanghai China Sex Worker and MSM Center.

^c^MSM: men who have sex with men.

#### Program Behavioral Skills Delivery

##### Individual Skills Training

On the basis of the previous steps of IM and co-design sessions (see steps 2-3), the content of individual skill learning includes the following modules and is delivered in the form of podcasts over the course of 5 weeks: (1) psychoeducation of emotions: the process of emotion response, types of emotions, emotional intensity, and functions of emotions; (2) how to increase tolerance of distress and get through a crisis situation; (3) how to accept reality and relieve emotional suffering; (4) how to regulate emotions through change behaviors and how to reduce overall emotional vulnerability; and (5) summary of all skills and highlights of takeaways.

##### Group-Based Skills Training

The 5 weekly group-based skills training sessions are parallel to the individual skills learning on the app. Program users will first learn the content on the app each week before attending the weekly skills groups (3-5 group members) facilitated by trained SCMC staff. In the skills group, the topic of each skills training session is the same as the content covered by the app in a given week. The skills group will have an onboarding meeting (1) to orient the group members to the purpose of skills training, introducing the skill deficit model of emotional and behavioral dysregulation and outlining the content covered in this intervention; (2) to introduce the group format and guidelines, including the roles of the skills trainer (ie, SCMC staff) and the group members; (3) for group members and SCMC staff to get to know each other; and (4) for individual group members to share their personal goals through this intervention, including behaviors to reduce and skills to increase.

The skills training group structure was adapted from the standard DBT skills training. The weekly session lasts approximately 1.5 hours and comprises the following components: (1) mindfulness practice (5-7 min), (2) summary of the skills covered by the app in the week before this session (5-10 min), (3) homework review (45 min), (4) preview of the following week’s content (5-10 min), and (5) assignment of a worksheet (to practice the learned skill on a given week) for the following week and goal setting for the following week (5 min).

The homework aims to help program users identify opportunities in their day-to-day lives. The content of each homework assignment was designed to match the skills learned on a given week. For example, in the week in which individuals learn distress tolerance skills, their homework will guide them to describe a situation in which their distress was high, note down their distress level before and after skill practice, and reflect on how well it went and areas for improvement.

The purpose of the skill group is to provide a supportive environment at the community level for peers to learn and practice skills together through peer and SCMC staff modeling. The group members will take turns to share how they practiced the skills during the week, and the rest of the group members and the SCMC staff will provide validation and constructive feedback. If the individual encounters any problems with applying the skills on their own, the group will help the individual problem-solve and provide constructive feedback as needed. The norm of attending groups regularly and practicing skills between sessions established through the skills group can motivate people who conform to this norm and increase their probability of practicing skills between sessions. This allows group members to interact with people with similar struggles, and the development of a validating and supporting environment is therapeutic for all members. Stickers will be given out to those who attend the skills group and practice skills that week [37].

Adapted from the DBT skills training group guidelines, the skills group guidelines serve as a structure that outlines a set of behaviors and principles that group members are expected to follow while in the skills group:

1. Participants in the skills training group support each other by preserving confidentiality, attending groups regularly, validating others, practicing skills in between sessions, providing noncritical feedback, and opening up to receive help from other group members or SCMC staff.
2. Participants need to call ahead of time to let the SCMC staff know if they are going to be late or miss a session.
3. Participants need to be aware of how dysfunctional behaviors such as self-injury and substance use are discussed in the group given that others may be influenced to engage in these behaviors. The mention of these behaviors should be brief and the focus should be on skills practice or problem-solving around the behavior.
4. To prevent interpersonal conflicts from complicating the group dynamic, participants can obtain the contact information of other group members and form supportive peer relationships outside the group after completing the study period.

##### Phone Coaching

In addition, the same SCMC staff trained to facilitate skills groups will be available for on-demand between-session skills coaching via phone call or SMS text message to help participants identify and practice skills in difficult situations and get through crisis situations when emotional pain is intense and dysregulated behaviors may occur as a result. It is common for individuals to have difficulty asking for help. Some contributing factors may be not being aware of the situations in which they might need help, failing to ask for the type of help they need, or asking for help indirectly (eg, hinting). Phone coaching enables the SCMC staff to cultivate individuals’ ability to effectively ask for help, provide feedback, and help problem-solve with ineffective help-seeking skills.

The general goals of phone coaching are (1) skills generalization, (2) validation of participants’ need for contact, (3) intervention on suicidal behavior (skill use and problem-solving), (4) crisis coaching (shaping them to context and using skills before the problem behavior occurs), and (5) contingency management.

The general guidelines for phone coaching include the following:

1. Validate and reinforce skillful help-seeking behaviors, such as clearly stating the situation, what they have tried, and what they need more help with.
2. Generally focus on how to use skills to prevent rather than manage a crisis when it occurs so that the frequency of phone coaching will reduce over time.
3. Keep the phone coaching session to between 5 and 15 minutes and use a directive communication style given the limited time.
4. Decrease crisis behaviors, increase skill generalization, and reinforce effective skill use.

#### Program Technological Delivery

We used the BIT model to guide the design and report of the technological delivery of the intervention, including elements, characteristics, and workflow [19]. The elements refer to the technical aspects of implementing the intervention content and behavior change strategies. The characteristics refer to how elements are presented, including the medium, complexity, esthetics, and personalization. The decisions were informed by co-design sessions and industry standards. The elements, characteristic considerations, and rationale are presented in Multimedia Appendix 1.

**Figure 2:**
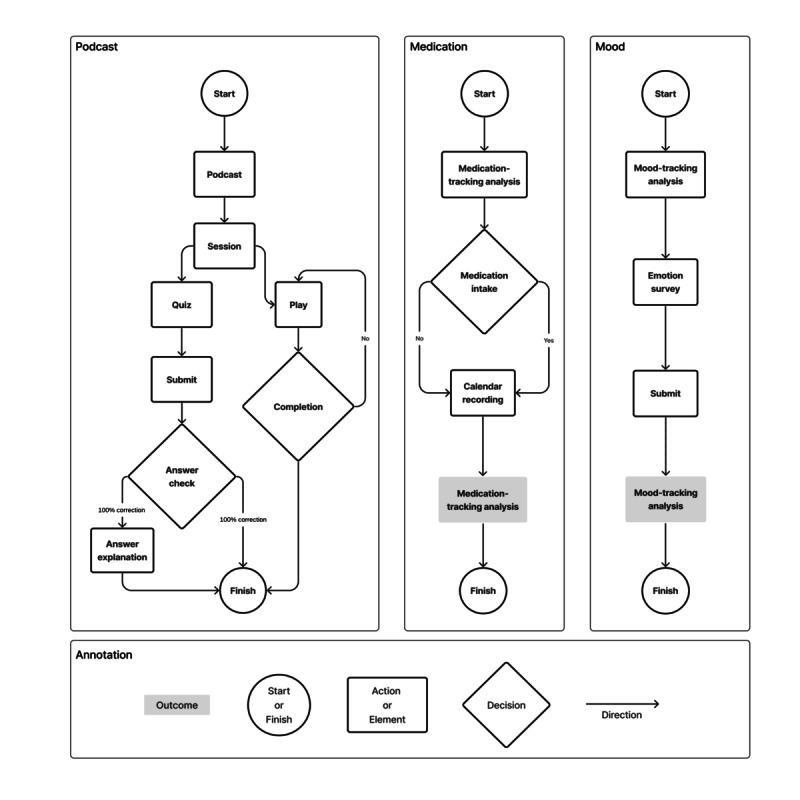
Intervention app workflow.

Workflow refers to the user’s interactions with the app to receive the intervention [19]. The mobile phone app was designed for users to complete weekly self-guided skills learning. The conditions under which elements of the intervention are delivered include time-based and task completion rules. New podcast content will be released at the start of every week. Users can decide when to access the intervention and can repeatedly listen to and review the content if needed (ie, individual-paced learning). Users need to finish listening to the podcast to access the quiz that helps consolidate their learning. The app content is delivered using a combination of hierarchical, tunneling, and matrix information architectures (Figure 2).

The hierarchical design of the home page presents all features and content of the app to the user in a top-down organization. Users will be taken through intervention modules following the tunneling design, where a step-by-step flow helps users navigate through the modules. The user has access to all the completed modules in a matrix design. The combination of these information architecture designs can achieve the following goals: (1) the hierarchical presentation of the home page is familiar to most users and provides a dashboard for all tasks and content; (2) tunneling optimizes the ordering of information delivery and, thus, intervention effectiveness and titrates the information presented to the user to reduce information anxiety; and (3) the matrix design allows for flexibility in accessing content [42,43].

## Discussion

### Principal Findings

This study illustrated the development of a DBT-informed intervention that integrated app-based individual skills learning, group-based skills training, and phone coaching to improve mental health outcomes for MSM recently diagnosed with HIV in China.

We used interdisciplinary theoretical frameworks and approaches, including behavioral science, clinical psychology, human-centered principles, and behavioral intervention technological delivery. Specifically, we used the IM protocol to guide the conceptualization of program goals and intervention development. The DBT model of emotion and emotional regulation skills informed the specification of performance objectives. The COM-B system of behavior and BCW further connected the performance objectives to intervention functions and strategies. Although the aforementioned theories informed the conceptual specification (conceptual how) of the behavioral intervention, the BIT model guided the technological delivery (technological how) of this intervention. The user-centered principles and strategies, as well as cultural adaptation considerations, were applied throughout all steps of intervention development to tailor behavioral components and technological delivery to the community.

Complex decision-making is involved in all steps of the development of interventions that aim to solve a specific health problem. This study presented a detailed intervention rationale that outlines the theoretical reasoning and behavior change mechanisms behind each intervention component. This is critical to the analysis of the active intervention ingredients in the subsequent feasibility and efficacy trial. A thorough understanding of the effects of each intervention component will aid future efforts to adapt and improve the intervention. In this study, we used the DBT model of emotion to understand the emotional difficulties and social and behavioral consequences revealed in the needs assessment stage. By mapping emotional difficulties from low to high emotion intensity, we determined the subsystems of emotion to intervene as our performance objectives, including reducing high emotional arousal, changing emotional expression to regulate emotions, and reducing emotional vulnerability. To achieve these performance objectives, we selected education, skills training, enablement, and modeling as the key intervention functions to affect capability, motivation, and opportunity of behaviors guided by the COM-B system and the BCW. The DBT treatment model of individual therapy, skills groups, and phone coaching then informed the operationalization of behavior change methods. There is growing evidence supporting the effectiveness of theory-based behavioral interventions [13]. IM offers a framework to synthesize conceptual (eg, DBT model of emotions and COM-B) and action (eg, DBT emotion regulation model and BCW) theories to derive a logical chain of decisions that determines the causal links between treatment components and outcomes [44].

In addition to theoretical considerations, this study also engaged community members and targeted users throughout development using user-centered design principles and strategies. The needs assessment with community members played an important role in determining the multilevel program objectives of this intervention, including individual skills learning and community-level capacity building. Staff training is an inherent component of this intervention to prepare the SCMC staff to facilitate skills training groups. Intervention testing and long-term sustainability hinge on a set of contextual limitations. Therefore, it is especially important to involve both intervention users and community partners in decision-making regarding intervention design to optimize feasibility, such as the length of the intervention, the intensity of staff training, and organizational support for staff to participate in the intervention study. The co-design sessions prompted community members to generate solutions to emotional difficulties (eg, come up with coping strategies regarding an emotional situation) and brainstorm services and intervention design (eg, individual format vs group format and on the web vs in person). This co-design process naturally incorporated the cultural adaptation considerations of DBT skills, including persons delivering skills training groups, metaphors and examples used in skills training materials, the use of existing cultural idioms to communicate DBT concepts, the method of using groups to complement app-based individual skills learning to reduce HIV- and mental health–related stigma, and the use of staff-delivered phone coaching to enhance effective skill generalization. The behavioral elements of this intervention went through iterative rounds of design and development through community engagement and consultation with experts to ensure appropriate translation of clinical and behavioral science into user-centered and practical intervention strategies. Many interventions developed by academic researchers have been mainly based on evidence from the literature and could benefit from end-user input early in the formative stage. The cycle from development to a randomized controlled trial for efficacy takes years, and more time is needed to transition to the effectiveness and implementation stage. A community-engaged and user-centered approach enables the research team to align their research goals with users’ needs and conduct iterative and rapid revisions to increase the contextual appropriateness, acceptability, and feasibility of the intervention and, thus, may significantly shorten the cycle from development to implementation and bring about a meaningful social impact faster. For example, the application of multiple frameworks such as multiphase optimization strategy and discover, design, build, and test synthesized the iterative nature of HCD principles and implementation of a forward approach to developing effective and scalable health interventions with rigor and efficiency [25].

This study used the BIT model to strive toward the synergistic effect between behavioral intervention content and technological delivery. In addition to the evidence-based behavior change strategies and coping skills, the effectiveness of behavioral health interventions hinges on operational factors such as the characteristics of the persons delivering them. We addressed this by providing training for SCMC staff who will facilitate the skills training groups as part of the intervention. The technological delivery of intervention functions (eg, education, training, and enablement) was guided by the BIT model. The characteristics of each technological element (eg, medium and complexity) were carefully determined based on design theories and standard practices to optimize the usability of the digital platform and create an engaging and pleasant user experience [19]. The combination of group-based skills training and app-based individual skills learning allows for easy access to digital content while providing peer support to enhance intervention engagement among users. In addition, user data collection provides valuable information on real-time user engagement with app content and the frequency of using a certain feature. For example, how long a user stays on a page or how frequently a user visits a page may indicate their interest level or need for specific content. Such information allows for future personalization of the app to tailor it to the user’s preferences and learning goals. User activity data can also be used to interpret intervention outcomes by isolating user engagement and adherence to the intervention as contributing factors toward the intervention outcome.

This study holds significant potential for improving mental health outcomes among MSM recently diagnosed with HIV in China, with the content and delivery of the intervention tailored to meet target users’ unique needs. Integrating app-based individual skills learning, group-based skills training, and phone coaching, the program aims to promote coping skills, foster community connection, counter feelings of stigma and isolation, and increase adherence to HIV treatment regimens among MSM recently diagnosed with HIV. The cultural adaptation considerations applied throughout the development process ensure a culturally sensitive care model that can effectively bridge the mental health service gap often encountered by this demographic. In addition, the findings of the future feasibility trial of this intervention could inform public health policy and serve as a template for similar initiatives in the future both in China and worldwide. Using mHealth technologies to deliver individual skills learning also allows for scalability, potentially affecting a broader population.

### Limitations

There are several limitations to this work. First, the needs assessment stage collected qualitative data and would benefit from quantitative survey data to evaluate the community service gap in mental health services and staff attitudes toward mental health training to provide such services. Second, personalization is still lacking in the current version of the mHealth app that delivers the individual skills training component. The user is able to mark their favorite coping skills for future reference, but the functionality of recommending similar skills to the user based on the user-reported problem situation is yet to be developed. Given the highly contextual nature of coping skill use, the next version of this app aims to use computational models to account for the dynamic interaction between individual behavior change progress and situational factors. Third, this study used co-design sessions as a user-centered design strategy to engage end users in designing the intervention. At the same time, the extent of user engagement in our study fell on the lower end of the participatory design spectrum for intervention delivery design because of limited exposure to mental health services among the study participants. Approaches such as using a user journey map can engage end users in actively conceptualizing the overall intervention program. Fourth, our ability to engage the community in a co-design session may be hindered by the limited interactions that can occur within a brief time frame. Future iterations of intervention design and improvement could adopt a longer participatory design process by following up with the participants over several months to build rapport with them and understand their needs. For example, users can participate in the cultural adaptation of DBT-based skills by cocreating intervention materials such as culturally informed metaphors. In addition, we made modifications to the structure of the last 3 co-design sessions after realizing that the participants had difficulty distinguishing between coping strategies for low- and high-intensity emotions. We started the co-design session by asking participants to rank emotion intensity first before asking them to generate coping strategies. Finally, our usability testing collected feedback from users, which generated valuable design insights. Although we were not able to implement every design solution in the current version of the app owing to timeline constraints, we implemented changes that were important to the user experience and effective delivery of our intervention content in the current version of the app.

### Conclusions

We designed a DBT-informed intervention that aims to improve mental health outcomes for MSM recently diagnosed with HIV in China using a systematic approach that may serve as a blueprint for future interventions. The detailed description of the theoretical basis for intervention development will aid the identification of active intervention ingredients in future trials. The content of the individual skills learning can also be easily adapted to help a broader group cope with similar acute and chronic emotional struggles. The IM framework informed by user-centered design principles and cultural adaptation considerations offered a systematic approach to develop and tailor the current intervention. Specifically, the DBT model of emotion, COM-B system of behavior, and BCW informed the conceptual mechanism of the behavioral intervention. The BIT model facilitated the translation of behavioral intervention strategies into technological delivery components (ie, the technological mechanism). Future research, currently underway, will pilot-test the intervention to gauge acceptability; feasibility; and, ultimately, efficacy and effectiveness.

## Appendix

Multimedia Appendix 1 Technology delivery considerations guided by the behavioral intervention technology model.

## Abbreviations

BCW: Behavior Change Wheel
BIT: behavioral intervention technology
COM-B: Capability, Opportunity, and Motivation–Behavior
DBT: dialectical behavior therapy
EVF: ecological validity framework
HCD: human-centered design
IM: intervention mapping
mHealth: mobile health
MSM: men who have sex with men
SCDC: Shanghai Municipal Center for Disease Control and Prevention
SCMC: Shanghai China Sex Worker and Men Who Have Sex With Men Center

## Data Availability

The data generated during this study are not publicly available to protect participants from the potential impact of HIV- and men who have sex with men–related stigma in China. However, the data are available from the corresponding author upon reasonable request.
