# Supplementary material for: Enhancing Mental Health and Medication Adherence Among Men Who Have Sex With Men Recently Diagnosed With HIV With a Dialectical Behavior Therapy–Informed Intervention Incorporating mHealth, Online Skills Training, and Phone Coaching: Development Study Using Human-Centered Design Approach
Source: JMIR Form Res. 2023 Oct 13;7:e47903. doi: 10.2196/47903 (PMC10611999; doi:10.2196/47903)
Supplement: Multimedia Appendix 1 [file formative_v7i1e47903_app1.docx]

**Multimedia Appendix 1.** Technology delivery considerations guided by the behavioral intervention technology model.

| Element^a^ | Description | Characteristics | | | |
| --- | --- | --- | --- | --- | --- |
|  |  | Medium | Complexity | Esthetics | Personalization |
|  | | | | | |
| Information delivery | Each module consists of a 3-minute mini lecture and a 10-15–minute mock therapy conversation between a therapist and a client on the topic of each module. At the end of the mock therapy, the mock therapist briefly summarizes the important takeaways from the session and leaves homework for the client. In addition, the program user will complete a quiz (4-5 questions) after each module to consolidate learning and enhance engagement with the intervention content. | We chose to use a combination of audio and text (content outline) to deliver the module content of coping skills. Specifically, each module will be published as a podcast accompanied by an outline of the podcast content. The media richness theory suggests that forms of media vary in their ability to convey different forms of information. The information delivered is most effective when the communication channel and media aligns well with the goal of the intervention and user capabilities^b^. On the basis of the PD^c^ sessions, we chose audio format over video. Although both can effectively communicate the module content by presenting lectures and mock therapy conversations, audio format eliminates unnecessary visual distraction, creates space for users to freely follow the content outline, and is easily accessible when mobile data are limited (eg, during transit). In the co-design session with participants, audio, video, and images were perceived as more engaging than text only; although multiple delivery formats (audio and video) were perceived as more engaging, simplicity and ease of access were also highly valued. | We kept the complexity of the information relatively low to make it easily accessible to users of all educational backgrounds. Specifically, we used everyday language and carefully removed jargon in both the didactic and mock therapy components. The mock therapy is also structured to make it easy to follow. The quiz is presented in multiple-choice format so that users do not feel overwhelmed by text entry tasks. | Color considerations: with regard to the consistency and accessibility of the design language, thorough consideration was given to both style and color. A thorough examination of various color schemes was conducted, ultimately leading to the determination that an analogous color scheme aligns with the theme of hope and support. To ensure legibility and comply with the Adobe Web Content Accessibility Guidelines, the colors pink and dark violet were chosen. Simplistic design: we simplified the features and user flow for less distraction and interference to reduce mistakes and confusion and increase overall usability and accessibility. | The following aspects were considered based on the cultural adaptation framework: language, persons, metaphors, content, concepts, goals, and methods. |
| Logs, reports, and visualizations | Users track their medication taking using a calendar. Weekly mental health symptoms are tracked using a survey. | Data from medication and mental health logs will be presented to the user in the form of a bar graph and line chart on a weekly basis. Research suggests that tracking behavior can increase motivation for medication adherence. Building mental health awareness through tracking is the first step toward identifying places to use coping skills. | We designed the medication-tracking feature with a minimal number of clicks required for users to mark their medication as taken. Through the automatic display of a pop-up upon accessing the medication-tracking page, users are able to quickly and easily mark their medication with a single click. To indicate in which days the medication has been taken, we used a simple color scheme with the theme color for taken medication and a blank space for medication not taken. We incorporated a toggle switch that allows users to switch the status of medication taken on current or previous dates. Regarding data visualization, we included different charts to intuitively display the information and encourage users to be aware of their progress. We used a line chart to demonstrate the trend of mental health status and a bar graph to visualize the count data (number of days in which medication was taken during a week) to emphasize the importance of taking medication on a daily basis. | In our medication-tracking visualization (bar graph), we used a color-coding system with red (1-3 days per week), yellow (4-5 days per week), and green (6-7 days per week) to indicate different levels of medication-tracking adherence. These colors were carefully selected by our design team to align with industry standards and convey the necessary information without appearing alarming to the user. A similar color-coding system was used to indicate the results of the user’s depression tracking based on their scores on the PHQ-9^d^. The color spectrum used for this visualization ranges from red (severe) to dark green (nonminimal), with orange (moderately severe), yellow (moderate), and light green (mild) in between, providing users with an easy way to understand the level of depressive symptoms. | —^e^ |
| Notifications | We use within-app notifications to prompt the user to listen to newly released podcast content, log medication daily, and log mental health symptoms weekly. | A colored dot on the app component indicates new content or incomplete tasks (eg, medication and mood tracking). Notification messages are pushed to the user’s WeChat chat page for new podcast content and reminders for completing tasks (quiz). | The notification dot and messages have a low level of complexity. The dot only appears when there are unfinished tasks to remind the user to complete them. The messages sent to the user’s WeChat will consist of a brief headline and a concise description of the content so that the user is aware of the incoming information. | The color and size of the notification dot follows the guidelines of WeChat notifications to reduce users’ cognitive burden by maintaining consistency with industry standards. | — |
| Passive data collection | We will collect user activity during app use, such as frequency of clicking and listening to a podcast; complete rates of podcast; time spent on the quiz; and clicks into the calendar, mood-tracking, and visualization pages. How long a person stays or how often they visit a page might indicate their interest or their need (or lack thereof) for specific content or features. The number of visitors to certain pages similarly reflects the importance of certain features or content. The aim is to assess user engagement with the intervention content and the tracking features of the app for future iterations of app feature development and personalization of the app. | Statistics of number and location of clicks, user data of listening length, and frequency and time spent in each page are collected and stored. | Users’ data are collected in the back end and visualized in appropriate graphs to indicate engagement with app features for researchers to uncover insights. For example, the number of visitors to each page within the app is visualized using a bar graph to reveal insights regarding the popularity of certain content. The number of daily visitors to the app is visualized using a line chart. The number of times a page on the app was shared by users can also be tracked. | The esthetics of visualization of user activity data follow the WeChat UI^f^ guide. For example, all graphs use green as the main color, similar to most elements on WeChat. Red is used to indicate negative change (eg, decrease in the number of visitors to a certain page), whereas gray is used to indicate no change. | These data are examined to give researchers information about how people are using the app, including any issues or errors that come up and what people are doing. For example, how long someone spends on a particular page might tell researchers whether that person was able to find a specific button easily. In addition, how long a person stays or how often they visit a page might indicate their interest or their need (or lack thereof) for specific content or features. The aim is to assess user engagement with the intervention content and the tracking features of the app for future iterations of app feature development and personalization of the app. |

^a^This app is embedded in WeChat (ie, WeChat mini program). Thus, the overall design esthetics need to follow the guidelines published by the WeChat user interface guide.

^b^Ishii K, Lyons MM, Carr SA. Revisiting media richness theory for today and future. *Hum Behav Emerg Technol* 2019;1(2):124-131. DOI: 10.1002/hbe2.138.

^c^PD: Participatory Design.

^d^PHQ-9: Patient Health Questionnaire–9.

^e^Not applicable.

^f^UI: user interface.
